# Brain-Computer Interface tool use and the Contemplation Conundrum: a blueprint of mental action, agency, and control

**DOI:** 10.1093/oons/kvaf002

**Published:** 2025-06-11

**Authors:** Dvija Mehta

**Affiliations:** King’s College, University of Cambridge; Leverhulme Centre for the Future of Intelligence, University of Cambridge, 16 Mill Ln, Cambridge CB2 1SB, Cambridgeshire, United Kingdom

**Keywords:** Neurotechnology, ethics, mental action, Implementational control, overt actions, agency, intention, imagination

## Abstract

This paper approaches the role of intentional action in brain-computer interface (BCI) tool use to allow for an ethical discourse regarding the development and usage of neurotechnology. The exploration of mental actions and user control in BCI tool use brings us closer to understanding the philosophical underpinnings of intentions and agency for BCI-mediated actions. The author presents that under some theories of intentional action, certain BCI-mediated overt movements qualify as both voluntary and unintentional. This plausibly magnifies the ethical considerations surrounding BCI tool use. This problem is referred by the author as the contemplation conundrum. Thus, the paper proposes research scope for the neural correlates of intention formation and the neural correlates of imagination aimed at clarifying implementational control and safeguarding privacy of thought in BCI tool use.

*“While a BCI can easily detect neural signals, the challenge comes from identifying relevant signals and processing them appropriately.”* [1]

## INTRODUCTION

Recent advances in neurotechnology – such as brain–computer interfaces (BCIs) and intracranial neural implants – have renewed interest in questions of mind, consciousness, and *mental action*^1^ [3, 4]. At the same time, the rapid proliferation of these devices has opened a discourse on applied normative ethics [5]. Consider Neuralink’s first brain implant patient, Noland Arbaugh (hereafter ‘P1’), who can send commands to a computer purely via neural signals. P1’s intentions – such as moving a chess piece on a computer – produce overt changes in the world while bypassing bodily action. This case is especially compelling as it sits at the intersection of rich philosophical conversations on actions, mental acts, agency, and implementational control.

In light of this, the central question I address is *whether all BCI-mediated visible*^2^  *movements qualify as intentional actions of the user.* Accordingly, I draw on Mele’s causal–teleological theory of intentional action—which requires both a content condition (intention must accurately represent the movement) and a guidance condition (implementational control over execution) [6]—and on Searle’s causal account of intention, which grounds how an intention functions as the proximate cause of action and underpins moral responsibility [7].

Answering this question is pivotal for ascribing agency and responsibility of actions. In what follows, I introduce the contemplation conundrum, which shows that because BCIs rely on user’s intended imagination—i.e. *motor imagery*^3^—engaging both visual and kinaesthetic neural pathways [8, 9]—the device may register any of several overlapping imaginings rather than the agent’s singular intended movement, thereby undermining the user’s implementational^4^ control and yielding voluntary movements that don’t satisfy the conditions of intentional actions upon some theories of action ([6, 7]). From this, the core hypothesis emerges: *if certain BCI-mediated movements are unintentional, albeit falling under the umbrella of a voluntary overt action, then they pose distinct ethical challenges.*

Beyond conceptual inquiry, the impending commercialization of BCIs demands urgent ethical scrutiny. Examining BCI tool use under the paradigm of intentional action deepens our understanding of how agency is constituted by intention—and, crucially, how BCI-mediated movements diverge subtly from paradigmatic bodily actions, complicating attributions of moral responsibility [1, 10].

Arbaugh’s N1 implant exemplifies this dynamic: his every command—whether cursor movements or chessplay—derives solely from imagined motor imagery, there is no bodily movement involved, not even via a prosthesis. Historically, BCIs have restored communication and basic control for patients with diminished motor function, limb loss, paralysis, or locked-in syndrome. And BCI-driven prostheses translate users’ motor imagery directly into limb movements [11]. In each case, the BCI functions as a mediator between covert intention and overt effect.

In this paper, I take Arbaugh’s case as a central discussion point to sketch a conceptual blueprint of action, agency and implementational *control* in advanced BCI use. To date, little philosophical literature has addressed the specific interplay between neural processes and mental acts of imagining, especially in BCI tool use. Consequently, this blueprint is not definitive but serves to map the terrain of this emerging field and to catalyse further philosophical and ethical exploration.

In Section 1, I unpack the distinction between basic and non-basic mental acts [12] using Mele’s and Searle’s accounts of intentional action ([4, 6, 7]), showing how *covert* mental actions constitute *overt* publicly observable BCI-mediated actions. This further explains how a BCI-mediated action is attributed to both the user and the tool. In section 2, a deep-dive into the intentionality of covert acts illustrates how overlapping motor imagery can undermine implementational control and produce voluntary yet unintentional outputs – this central problem is what I call the contemplation conundrum. Finally, section 3 reframes these issues as an agenda for future research—outlining the empirical studies and theoretical refinements needed to clarify the neural correlates of intention formation and imagination, to improve BCI design, and to safeguard both agency and thought privacy.

## SECTION 1: ACTIONS, INTENTIONS AND PROBLEMS OF AGENCY & CONTROL IN BCI USE

In late March 2024, a livestream demonstrated P1 playing chess on the computer simply using his thoughts. After 8 years of living with paralysis, P1’s brain implant allowed him to regain certain capabilities previously inaccessible to him, like controlling a cursor on the screen. Although a leap for P1, doing things with thoughts comes with a trail of philosophical and ethical queries, especially the question of whether the patient has agency over BCI-mediated overt actions.

The understanding of action in BCI tool use and its implications remains under-explored still, although Steinert et al [10] and Rainey et al. [1] provide an insight into this area. Steinert et al’s paper addresses the question of what types of theories of action can be applied to BCI tool use, and they adopt a disembodied agency approach to answer questions of the patient’s sense of agency and sense of ownership of BCI-mediated actions. Such a view is more concerned to explain how the user *experiences* something as an action rather than when it *really is* an action. To grasp the latter, we need to move beyond the phenomenology of action to the underlying conditions of something being an action. Thus, I rather propose the adoption of an intentional theory of action. Such a theory advocates that moral responsibility is primarily determined by the agent’s intention rather than the outcome of their bodily movements, emphasizing that intentions are self-referential and act as the cause leading to an effect.

Active BCI tool use is vastly intriguing because of the technology’s heavy reliance on the user’s mental states, i.e. intentions to bring about effects in the world. Due to this, an intentional approach provides a mechanistic lens to view visible action execution in BCI tool use to then answer questions of *agency*. Beyond active BCIs, other types exist, such as reactive and passive systems. Steinert et al. [10] provides a comprehensive study of these categories, wherein reactive BCIs rely on the user’s involuntary brain responses to external stimuli, such as event-related potentials, to initiate an action or response. In contrast, passive BCIs do not facilitate action directly but instead monitor the user’s mental states, such as stress or fatigue, to adapt a system or environment accordingly.

Each type of BCI tool presents unique ethical and practical considerations. For instance, while active BCIs emphasize intentionality and agency, reactive BCIs raise questions about the interpretation and control of involuntary responses. Similarly, passive BCIs highlight concerns about privacy and consent, given their focus on continuous monitoring of mental states. Together, these types illustrate the diverse and complex ways in which BCI technology intersects with human cognition, agency, and ethics, broadening the scope for further research and exploration. In this paper, I focus on active BCIs, where users intentionally produce brain activity for the BCI to decode and process.

Therefore, first forming a deeper understanding of visible BCI mediated actions is crucial to understanding the ethical consequences of active BCI usage. This will set the stage for an in-depth analysis of *responsibility* and control of said actions in section 2.

### Types of actions

#### Overt and covert

According to law and policy, agency and responsibility for actions are typically assessed through overt actions—visible actions that cause changes in the external world and are paradigmatically associated with bodily movements, such as raising an arm or picking up a pen. However, the law also places critical importance on the mental states underlying these actions, particularly intention, which plays a central role in determining culpability through the concept of *mens rea* (guilty mind). For instance, the distinction between intentional and unintentional actions, such as murder versus manslaughter, highlights the legal emphasis on assessing intent. While covert acts, such as forming an intention or making a decision, are not typically actionable on their own, they gain relevance when they result in overt actions or provide evidence of intent, as in cases of conspiracy or attempted crimes.

This distinction between overt and covert actions becomes particularly significant in the context of BCI tool use, where the connection between mental states and physical outcomes is mediated by technology. In traditional contexts, overt actions are directly observable and tied to physical bodily movements; however, in BCI-mediated actions, overt outcomes might not directly correlate with the user’s intentions. For example, a patient using a BCI to control a robotic arm might accidentally trigger unintended movements due to noise in the neural signals or limitations of the system. While the physical movement of the robotic arm is overt and observable, the patient’s mental state—such as whether they intended the specific movement—remains covert and accessible only through indirect inference.

This divergence raises critical questions about how responsibility and agency are assigned in BCI-mediated actions. If overt actions resulting from BCI use do not align with the user’s intentions, should we treat such actions as unintentional despite their observable outcomes? Moreover, covert actions such as forming an intention to act or controlling focus during BCI tool use take on an amplified importance, as they provide the foundation for the system’s operation. Unlike in traditional contexts, these covert acts are no longer entirely internal—they are translated into physical actions through the BCI system, blurring the line between internal mental states and external bodily movements.

To address these complexities, Rainey et al. [1] propose amendments to law and policy, suggesting that the concept of ‘willed bodily movement’ be expanded to account for cases where overt actions are mediated by technologies like BCIs. This proposal emphasizes the need to incorporate covert mental acts more explicitly when evaluating agency and responsibility in BCI-mediated contexts. Such considerations challenge conventional legal and philosophical understandings of action, prompting a re-evaluation of how we define and assess both overt and covert actions in scenarios where technology serves as an intermediary between mind and body.

#### Voluntary and involuntary

Voluntary actions are those performed with conscious control —that is, willed acts over which an agent determines the ‘if,’ ‘when,’ and ‘how’ of execution, thereby exercising both executory and implementational control (see Section 1.4). For instance, when John turns on a light to brighten a room, his intention directly guides the bodily movement required to flip the switch. In contrast, involuntary actions—such as blinking or breathing—occur without conscious initiation, awareness or control. Rainey et al. [1] focus on this distinction and argue that some BCI-mediated overt actions may fall outside users’ implementational control and therefore resemble involuntary acts. In Section 2, I extend this distinction by considering how certain BCI outputs can be voluntary yet unintentional.

#### Intentional and unintentional

This brings us to a further distinction between voluntary and involuntary actions: the distinction between intentional and unintentional actions. A goal-oriented action performed by an agent with a purpose qualifies as an intentional action. Intentional actions involve control and foresight into their consequences. For example, John turns on the light because he intends to brighten the room, and he knows that flipping the light switch will achieve this result.

Actions may qualify as intentional under one description but fail under another. Suppose John intends to brighten a room and flips a switch, but the connected heating system activates instead. Under the description ‘flipping the switch’ or ‘attempting to turn on the light,’ his act was intentional; under ‘turning on the heating,’ it was not. Philosophers such as Anscombe [7, 13], and Mele [6] invoke this descriptive relativity to clarify how intentionality depends on matching an agent’s intention to the action’s outcome. In this paper, I argue that some BCI-mediated movements, although voluntary, may similarly fail to satisfy the correct content conditions of intentional action.

In this paper, I argue that some BCI-mediated movements, while voluntary actions, could also lack intentionality under certain descriptions. This adds another layer of complexity to the problem of control and, consequently, agency. Such scenarios necessitate a deeper examination of the role of intentions, which are covert acts, in establishing where agency should be placed and in understanding how much control a BCI-integrated agent truly has over their overt public actions.

### BCI tool use and action

Steinert et al. [10] argue that every BCI-mediated act qualifies as a **non-basic action**, since it is performed by means of a distinct covert act rather than by direct bodily movement. Following [12], a **basic action** is one executed directly (e.g. John raises his hand), whereas a **non-basic action** is performed by means of that basic action (e.g. John waves to his friend by raising his hand).

For a BCI-integrated user, every visible action is constituted of a covert basic act. In this case, a BCI user’s covert basic act is **motor imagery**—the imagining of a movement—which the interface decodes and instantiates as an overt effect. Consider a prosthesis user ***W,* for whom raising his prosthetic is the same as it is for John.** To raise his prosthetic arm, ***W*** must first imagine the arm rising (a basic mental act); that imagining then constitutes the prosthesis’s elevation (a non-basic overt act).

P1’s case is subtly different than *W* because there is no bodily motion or prosthetic intermediary. Instead, his imagined cursor trajectories function as the covert act that produces visible on-screen effects. It is relevant to focus on the P1 case because in the absence of any peripheral movement or prosthetic intermediary, the only locus of his agency lies in his covert intention-formation and motor imagery.

As P1 himself notes *‘it just became intuitive for me to imagine the cursor moving… I just stare somewhere on the screen, and it would move where I wanted it to.’* I emphasize here on the word ‘*imagine*’ because BCI tool users are often asked to imagine the actions they aim to bring about into the world. BCIs operate by detecting neural activity tied to the user’s intent—specifically, motor imagery, or vivid mental representations of movement—and translating those signals into commands for external devices. Neurophysiologically, BCIs isolate task-relevant activity—commonly in PPC, V1, and MT/V5—from background noise and then map these patterns onto device outputs [11, 14]. More vivid motor imagery strengthens these neural signals, thereby enhancing decoding accuracy.

But the shallow use of semantically rich terms such as ‘intent’ and ‘imagining’ leads to complexity. These terms carry nuanced meanings that when used superficially can contribute to misunderstandings and skin-deep analyses of complex phenomena. In particular, ‘intent’ is multifaceted: a user may entertain multiple concurrent imaginings, each constituting a discrete intentional state. Without a clear delimiter, we cannot know which covert act the BCI ultimately enacts. Section 2 below develops the ***contemplation conundrum****,* demonstrating how overlapping motor imagery can erode implementational control and yield voluntary yet unintentional actions. Before turning to that analysis, we first examine how covert actions constitute overt actions through intentions.

### Mental processes and doings

Having established the importance of covert actions in BCI tool use, we must now unpack the **causal chain** linking mental states to overt actions. To do so, I provide a conceptual distinction in *mental processes* and *doings*.

Mental processes encompass our beliefs, desires, mental *affordance*s^5^, wants, intentions, and imaginations [2, 15]. These can form mental states derived from creating mental representations of external affordances, desires or even internal voluntary mental acts of imagining, as well as involuntary instances of mind-wandering. Mental processes can be a cause for an effect in the world. This effect is usually an event that occurs, i.e. a doing: the event mental processes bring about in the world [12]. In other words, *doings* are *mental processes that are acted upon*. John’s intention to wave (mental process) realizes as the physical gesture of his hand waving (overt doing). In addition to this, a mental act can also be a doing, although this is a covert action, but if the content of a particular intention is fulfilled by means of a mental act, it can qualify as a doing, or a mental doing. Like in the case of recall or intended imagination. This mental act is crucial to this paper because BCI integrated users are expected to perform the mental act of imagining their intent to constitute an overt action.

Doings can be either voluntary or involuntary, they are intentional and unintentional. But when an overt doing and a mental process coincide by means of an intention, they form a voluntary intentional action. Like in the case of wanting to turn on the lights. John’s intention to turn on the light was fulfilled by means of him raising his hand and flipping the switch. This is a voluntary action because John has control and willingly performs this action of flipping the switch. His intention of brightening the room is fulfilled when he flips the switch^6^, which makes this a voluntary intentional action. In such cases, the intentional mental state is inseparable from the doing. To unravel this, I draw upon Searle’s prior intention and intention-in-action approach.

This further allows me to break-down the causal chain of action in BCI tool use and explore the question of *whether all BCI integrated overt actions are tied to the patient’s agency.*

In BCI contexts, this chain inserts an intermediate covert act:

1. **Intention X**: the user forms a mental intention (e.g. ‘move cursor right’).
2. **Motor Imagery M**: X triggers vivid imagining of the movement, engaging visual and kinaesthetic pathways [8, 9].
3. **Overt Effect Y**: the BCI decodes M into a device command, producing the cursor movement Y [11, 14, 16].

Formally, X ⇒ M ⇒ Y.

In paradigmatic bodily action, the chain **X ⇒ Y** is direct, clearly satisfying both Mele’s content and guidance conditions [6]. Under BCI mediation, however, the extra step M allows for alternative motor-imagery states M′—any of which might be decoded—so that willed actions can fail to meet the correct content or control conditions. Empirical work shows that decoder drift and the overlapping neural substrates of prediction and intention further destabilize this mapping over time [17, 18].

This fragility—where covert and overt acts coincide only by means of a contested covert imagining—sets the stage for the **contemplation conundrum** (Section 2), in which overlapping mental processes undermine implementational control and yield voluntary yet unintentional outputs.

This analysis brings us back to the question of whether all BCI-mediated actions qualify as intentional. Below, I therefore examine Searle’s distinction between **prior intentions** and **intentions-in-action**, showing how these concepts bear on agency and responsibility in the BCI context.

### Prior intentions and intentions-in-actions

Searle provides an important distinction in how intentions and actions relate to understand mental acts qua covert acts. Covert actions can be either basic or non-basic. Non-basic covert actions involve prior intentions or intentions-in-actions to allow for doing.

Deciphering this is fundamental in understanding the significance of intentions in covert and overt actions, particularly in cases where voluntary overt actions may still be unintentional. In the context of BCI tool use, where neural activity mediates physical outcomes, it is insufficient to assume that a voluntary overt action automatically reflects agency. Instead, the satisfaction of the intention underlying the overt act determines whether the user has implementational control over their actions. Rainey et al. [1] explore how implementational control is often reduced in BCI-mediated actions, as the interface may decode motor imagery that diverges from both prior and in-action intentions. Mele’s guidance condition [6] similarly requires that an agent oversee execution throughout. This highlights the importance of distinguishing between voluntary and involuntary neural acts—whether the contents of the intentions underlying the mental act of imagining are consistently satisfied in such scenarios.

Searle’s approach to intentionality grounds actions in intentions to account for moral responsibility. Within this framework, ***an action is intentional iff the contents of the intention are satisfied in the right way*** [7]. This avoids the problem of deviant causal chains, where an intended outcome occurs through unintended means. Intentions, according to Searle, are causally self-referential—they inherently refer to the actions they aim to bring about, linking the mental state to the resulting behaviour.

Searle’s causal theory of intention [7] distinguishes **prior intentions** and **intentions-in-action**. Prior intentions are formed before an action is performed and involve planning or deliberation. For instance, while reading this paper, you may form the prior intention, ‘I will read Searle after this.’ These intentions guide subsequent actions but do not directly execute them. In contrast, **intentions-in-action** occur in real-time, where the intention resides within the performance of the action itself. An overt example of this is me typing these words. There is no prior intention between the typing of each word, the intention simply resides in the act of me typing these words. Another example is John flipping a light switch to brighten a room, which exemplifies an intention-in-action—he acts directly without the need for intermediate deliberation. Similarly, a covert intention-in-action occurs when recalling what you ate for lunch, where the intention seamlessly integrates with the mental act.

This nuanced distinction between prior intentions and intentions-in-action provides a framework for analysing agency in BCI-mediated actions, where the alignment between mental states and overt outcomes is often complex and contingent on the system’s mediation.

**Figure 1:**
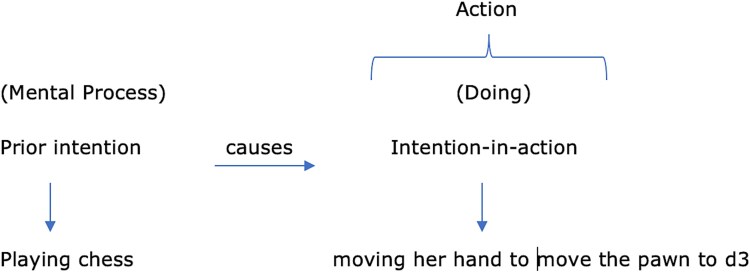
Cognitive-action sequence for a non-BCI chess move. [Diagram showing the mental affordance of the chessboard, formation of a prior intention, optional contemplation of candidate moves, and the intention-in-action that culminates in Lily physically moving the pawn to d3. Solid arrows indicate causal progression from covert mental states to the overt action.]

Let me present a case of a hypothetical woman Lily – not a BCI-integrated person – playing chess (see Fig. 1 below). Causally, she first perceives the chess board and has affordances of the same, i.e. the opportunities for action when she sees the chess board. These affordances are a mental process formed from pre-existing beliefs of interacting with an external object, i.e. the chess board. She then forms a prior intention, a mental state of knowing she will play chess. She may even contemplate what she will play, either involuntarily or voluntarily. After this, Lily simply moves the pawn to d3. This moving of the pawn is an intention-in-action because the intention lies within the act of moving the pawn. This moving of the pawn by Lily is a doing. Everything before this doing was a mental process. Perhaps if her contemplation was voluntary and intentional then that was a mental act. If it was mere mind-wandering akin with the affordance of the pawn and chess board, then it is a mental process.

Diagrammatically, one can present the Lily case this way to explain the mental processes leading to bodily action by accounting for her intentions:

Lily moving her hand to move the pawn to d3 is a non-basic action in the sense that her moving her hand constitutes the pawn moving to d3. In this case, Lily’s action is a voluntary intentional overt action because:

1. She wants to play chess, so she does.
2. The contents of her intention to play the d3 move were fulfilled when she moves the pawn.

Therefore, her intention of moving the pawn to d3 resides in her doing so. Her mental process of intention reflects in her doing. Thus, we can say that the overt action Lily performs can be tied to her agency since she is a goal-oriented agent that intentionally performed this move.

**Figure 2:**
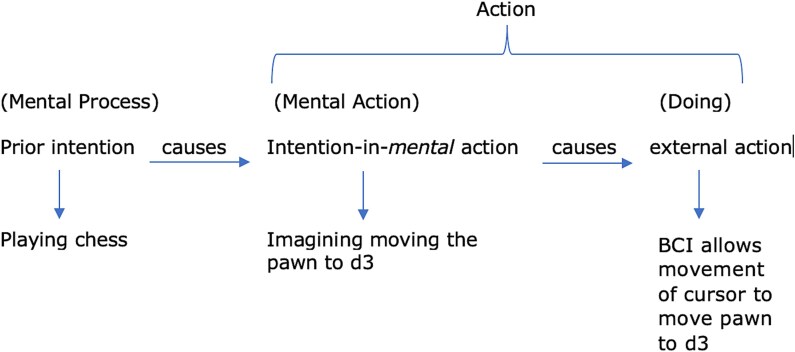
Cognitive action sequence for a BCI mediated chess move [P1 must intentionally imagine the pawn moving to d3 so the BCI may pick up this goal-oriented intention to decode, process and allow the doing of moving the pawn to d3. So, in P1’s case the intention of moving the cursor to move the pawn to d3 is not fulfilled by him merely imagining this happening. It is only fulfilled when the BCI picks up on this imagination to then decode the respective neural pattern, process and then produce an output]

Applying this to the P1 case, a BCI-integrated individual who intends to play chess on his computer (see Fig. 2 below). Causally, he would first perceive the chess board on the computer. He will have affordances of the chess board, same as Lily. Upon this he forms his prior intention to play chess. But he cannot simply move a pawn which would’ve been the case of an intention-in-action. He must rather imagine the move he wants to make, let’s again say he wants to move the pawn to d3. So he must now imagine the pawn moving to d3 which would be an intention-in-*mental*-action because this is a covert action. Note here that I add on to Searle’s prior intentions and intentions-in-action. I introduce the intention-in-*mental*-action, a crucial stage for BCI users. Therefore him imagining the pawn moving to d3, a mental act in itself, is constituted of his intention to move the pawn to d3. This mental act fulfils his intention to move the pawn to d3 (X)^7^. But there still remains the act of fulfilling the intention of actually playing chess on the computer (Y)^8^, which is thus mediated by the BCI. The movement of the cursor on the screen to move the pawn to d3 is the doing. Therefore the doing has fulfilled the contents of his intention to move the pawn to d3.

The voluntary intentional action here is thus attributed to P1’s intended imagination of moving the pawn + BCI mediated movement of cursor to move pawn.

*‘A successfully* ^9^  *performed intentional action characteristically consists of an intention in action together with the bodily movement or state of the agent which is its condition of satisfaction and which is caused by it. The account is extended to complex actions.’ [7].*

This sharing of the attribution of action in BCI cases raises the ethical worry of control. How much control does a BCI user truly have over their overt actions?

When it comes to the dimensions of control in actions, an important aspect that Rainey et al. [1] address is the shift from executory to implementational control. For example, in the case of a BCI user like P1, executory control might involve setting a goal to play chess, while implementational control requires the practical execution of the goal, such as imagining the movements necessary to control the cursor. This shift becomes complex with BCIs because the implementational control depends on the system’s ability to accurately decode the user’s mental imagery and translate it into action. Errors or delays in this process can result in a mismatch between the user’s intentions and the outcomes, raising ethical and practical concerns about the degree of agency the user truly retains. Executory control corresponds to general goal-setting, for example, P1 sets a goal to play chess; this is related to the ‘when’ and ‘why’ of an action. Implementational control encompasses the practical aspects of ‘doing’ the action; this is ‘how’ the action is performed [19]. For P1, this ‘how’ involves creating mental imagery of his ‘doing’. In regular cases, this is not a problem. But with BCI users, the implementational form of control is rather reduced due to the action being performed as a combination of the mental act of imagining + BCI-mediated movement. Somewhere between these two events, the dimension of implementational control suffers. I will come back to this when I present the contemplation conundrum in section 2.

One might say that as long as the covert action is intentional, the overt action will follow. Therefore, preserving the agency and control of the user. One may even say that the overt action here could be tied to P1’s agency as P1 forms a voluntary intention when he performs the mental act of imagining, thus arguing that P1 has control. But as I now present my arguments, it will get clearer that some covert actions in themselves are non-basic and carry layered intentions, thus questioning again the aspect of how much implementational control the user truly has.

### Dual-content intentions X and Y in P1’s case

Searle’s account requires that the **contents** of an intention must be fulfilled in the correct way for an action to count as intentional [7]. In P1’s BCI-mediated chess move, there are two distinct intentions, each with its own content:

*X-Int = t*he intention to imagine moving the pawn to d3, i.e. the intention for covert action.

*Y-Int =* the intention to effect the pawn’s advance to d3 on the screen, i.e. the overt cursor movement.

This is where the philosophical complexity of understanding intentions and actions gets amplified. In P1’s case, the added layer of imagining a said movement by means of creating mental imagery demands that P1 perform this covert act of imagining to create the imagery. Therefore, there exists the contents of this intention to perform the mental act of imagination, i.e. the covert act. Thus, it is plausible to say that X^10^ is the content of the intention to perform the mental act of imagining. Therefore to fulfil X, P1 must engage in this imagination. Only once **X** is fulfilled can the interface decode that imagery and produce the overt movement that fulfils **Y-Int**.

Formally:

*Premise1 (X-fulfilment)*: If P1 engages in the imagination of moving the pawn to d3, he fulfils contents X of this intention.

Upon doing this, his covert action is a successful intentional mental action.

But what about the intention to actually move the cursor on the screen to play chess? Let us take Y as the content of the intention to move the cursor to play the d3 move on the computer, so Y is the intention to perform the overt action.

But this intention can only be fulfilled if intention X is fulfilled.

*Premise2 (Y-fulfilment)*: If the BCI-mediated movement allows for movement of pawn to d3 overtly, then contents Y of the intention to play the move on the computer are fulfilled.

*Conclusion*: Both contents X and Y must be fulfilled for a successful voluntary intentional action.

Note here that the overt action is non-basic which means something constitutes it, i.e. the covert mental act. This is the ideal scenario, and if this is achieved then the voluntary intentional overt action directly reflects the patient’s agency. The fulfilment of the contents of both intentions, i.e. fulfilment of X and Y is causally self-referential to the agent.

An immediate puzzle arises at the moment of decoding: which of the two intentions—**X-Int** (the covert imagining) or **Y-Int** (the overt execution)—does the BCI actually register? Both intentions await fulfilment at time t = 1, yet neurophysiologically, no consensus marker yet distinguishes the neural signature of motor imagery from that of a distinct ‘intention to act’ [17, 20]. This indeterminacy signals a deeper complexity: what if the covert act **X-Int** itself is non-basic, comprising layered mental contents all awaiting fulfilment? To unpack this, I now focus on **X-Int** to expose its peculiar role in BCI-mediated action.

## SECTION 2: THE CONTEMPLATION CONUNDRUM

Coming back to the contents X and Y, another problem arises. Since X is the content for the fulfilment of a covert action, and Y for an overt action, it is essential to consider their interaction in the context of basic and non-basic acts. In this relationship, X often represents a foundational step—such as forming an intention or imagining an action—that causally leads to Y, which manifests as an overt action. The fulfilment of X is necessary for Y to occur, illustrating how covert acts underpin overt actions. What one gathers at this point is that X is a basic action and Y is non-basic. Causally, the covert constitutes the overt. So, the fulfilment of X constitutes the fulfilment of Y. But what if the covert act itself is non-basic? In such cases, it may involve multiple layers of content awaiting fulfilment, each corresponding to specific mental acts or processes. For example, the non-basic act of imagining a chess move includes basic acts such as contemplating potential moves and predicting their outcomes. These layered contents, collectively forming the non-basic covert act, highlight the complexity and interdependence of mental processes required to achieve a higher-order intention. To unpack this, I now focus on **X**—the covert mental act of forming an intended imagination—because in chess, imagining a move is itself a non-basic action.

So far, we have seen that covert acts are crucial for agency in BCI tool use and that BCI-mediated overt actions are attributed to the user+the tool due to the fulfilment of intentions that falls upon both. But, If every overt action followed directly from a single covert intention, ethical concerns would be minimal.

### Motor imagery overlap during contemplation

I now present the contemplation conundrum of BCI tool use to show that it is plausible for a BCI tool to perform a voluntary unintentional overt action, that does not fulfil the content of one of the intentions of the user. As I highlight in the previous section, the user has 2 intentions: X-Int and Y-Int at time t = 1.

Consider again P1’s remark: ‘It just became intuitive for me to *imagine* the cursor moving… I just stare somewhere on the screen, and it would move where I wanted it to.’

Echoing my earlier emphasis on ‘imagine,’ BCI users must mentally visualise the actions they seek to bring about, engaging both visual and kinaesthetic pathways [8, 9]. This reliance on motor imagery complicates agency and control because the interface must disambiguate overlapping or unintended neural signals—shifts in attention or effort can alter imagery’s neural signature, leading to misinterpretations [21]. Consequently, BCIs demand ongoing recalibration to distinguish genuine intent from background noise [9, 18]. Structured mental-training protocols can sharpen imagery fidelity and bolster implementational control [22], yet in the absence of proprioceptive feedback—such as in amputees—users may still lose fine-grained mastery over their imaginings, heightening the risk of unintentional outputs.

All of this stems from the fact that imagination engages motor-imagery networks across frontal motor-planning areas, V1, PPC, MT/V5, and inferior parietal regions [8, 9], generating neural patterns that BCIs detect once they exceed threshold^11^ levels. As Aflalo et al. [16] explain, ‘motor imagery recorded from populations of human PPC neurons can be used to control the trajectories and goals of a robotic limb or computer cursor.’

The contemplation conundrum asks the reader to consider the following possibility:

When P1 perceives the chess board and knows he will play chess, it is possible he engages in contemplations of the moves he could play. This could be something as simple as mind-wandering. But in situations like playing chess, the mind does more than merely wander [23].

Since playing chess is a complex task, the covert act of imagining the intended move in itself is a non-basic mental act [2]. This means that there are basic acts involved in formulating the intention to play a move. Several covert basic acts include thinking through moves or predicting the opponent’s response, this in turn accumulates into the complex, covert, non-basic act of forming an intended imagination of the desired move [23].

Therefore, the user’s mind cycles between potential moves (M1 & M2) and even imagine their outcomes. Suppose P1 first imagines advancing to d3, then within a fraction of a second switches to d4: rapid shifts in neural patterns will produce overlapping signals that the BCI must parse [21]. *Which of these contemplations would the BCI pick up on?*

Neuroscientifically, both covert consideration of move options and ‘intended imagination’ evoke overlapping neural activations across frontal motor-planning areas, primary visual cortex (*V1*), posterior parietal cortex (*PPC*), the middle temporal visual area (MT/V5), and inferior parietal lobule [8, 9]. Thus, when P1 *contemplates* potential moves, neural spiking occurs in all of these regions—just as it does when he forms an ‘intentional imagining’ of a move.

No known neuroscientific marker distinguishes one pattern of motor imagery from the other except the retrospective ascription of an ‘intention to act.’ Neurophysiological markers like the readiness potential—an EEG signal preceding movement by up to a second—have been shown to be indistinguishable from activity during mere motor imagery and to arise before any conscious intention, further confirming the lack of a unique neural signature for intention [20, 24].

Moreover, Bertoni et al. [25] demonstrate that the phase of low-alpha oscillations in primary motor cortex and supplementary motor area—occurring up to half a second before movement onset—predicts both explicit and implicit agency judgments. In other words, it is not only whether preparatory activity occurs, but when it aligns with endogenous rhythms, that modulates our sense of self-generated action. This concrete oscillatory evidence reinforces the absence of a unique intention signal and shows that BCIs decode motor-imagery features whose temporal dynamics are themselves entangled with agency.

The problem here is that BCI users are prompted to generate precisely that ‘intentional imagining,’ but there is not yet a signature for an intention separate from imagery. Empirically, we can pinpoint activations in regions associated with imagination—muscle control and movement in MT/V5, visualisation in PPC and V1—but we cannot isolate a marker that definitively signals ‘I intend to act.’ For example, Aflalo et al. [16] demonstrate how motor imagery in the PPC can be harnessed to control robotic limbs, while Schurger et al. [20] highlight the difficulty of isolating a neural event that corresponds exclusively to volitional commitment rather than preparatory imagery. These findings illustrate the intricate overlap between the neural correlates of intention formation and imagination, underscoring the difficulty of distinguishing between them in practical applications like BCI tool use. This conflation supports Schurger et al’s ‘brain+body’ model, which conceives action as the integration of mental processes and overt doing (as seen in section 1) rather than as a succession of separable neural events.

Without a clear neural delimiter for intention, it remains impossible to determine which mental event actually drives a given BCI-mediated effect—empirical research on the neural correlates of intention formation remains rudimentary [26]. Accordingly, BCI systems hinge on the user’s ‘attempt’ to move—that is, their *motor imagery* or *imagination*—and, following Aflalo et al. [16], any PPC activation representing a goal can be interpreted as an intention to act.

### Contents within X-int

Coming back to X being the intention to form an intended imagining: a non-basic act involving other basic acts as the contemplation conundrum posits. The contemplation conundrum highlights the difficulty of distinguishing between mental acts that contribute to imagining versus those that reflect an intention to act. It underscores the ambiguity in BCI contexts, where both types of mental acts generate similar neural patterns, complicating the process of determining which imagined scenarios lead to overt actions. This conundrum raises critical questions about agency and control in BCI-mediated actions. I ask the reader to consider the possibility that within the content X there exists Xα and Xβ. 


$$ {\textrm{Let}}\ \mathrm{X}=\{\mathrm{X}\alpha, \mathrm{X}\beta\}. $$

Where in, X = the content of intention to form an intended imagination to act (content of intention for covert action).

Xα = content of mental state to contemplate multiple potential moves (eg: pawn to d3 or d4) and outcomes.

and Xβ = content of imagining the specific, intended move (pawn to d3)


$$ \mathrm{X}=\mathrm{X}\alpha \cup \mathrm{X}\beta, $$

$$ \mathrm{X}\alpha \cap \mathrm{X}\beta =\varnothing $$

Therefore, for the non-basic covert action of forming an intended imagination of the user’s move, the basic acts of contemplation and deciding the move are involved.

Both Xα and Xβ contribute to imagination and motor imagery as seen in section 2.1. The BCI’s heavy reliance on the user’s imagination/motor imagery means the user may not have implementational control over which imagining the BCI acts overtly upon. In regular cases such as Lily’s, the executory and implementational control are evident. Lily can engage in these contemplations without the worry of a certain overt action coming to be unintentionally. Lily’s control over actions allows her to act overtly in an intentional manner. Say, Lily can contemplate between d3 and d4 but still act overtly to play d3.

P1’s additional X content means he may not as freely engage in X through Xα and Xβ. And let us not forget Y, i.e. the content of the intention to actually move the pawn (intention for overt action) which is constituted of X.

The ideal situation to allow for an intentional voluntary overt action is when the BCI acts upon the combination of Xβ + Y.

*Xβ + Y = voluntary intentional overt action.*

*Fulfilling Y makes this a voluntary overt act and fulfilling Xβ makes it an intentional voluntary overt act.*

Because BCIs decode whichever motor-imagery pattern crosses threshold—whether from Xα (contemplation) or Xβ (intended imagining)—it may instantiate **Xα + Y**, yielding a **voluntary but unintentional** overt action, rather than the desired **X***β* **+ Y**.

*Xα + Y = voluntary unintentional overt action.*

*Fulfilling Y makes this a voluntary overt act, but not fulfilling Xβ makes this an unintentional overt act.*

This possibility— *Xα + Y* —*is* the *contemplation conundrum*. It confronts us with a fundamental ethical dilemma: if the BCI acts on layered imaginings rather than the user’s true intention, how can we ascribe moral responsibility? And practically, this complicates the development of more precise BCI systems capable of differentiating between mental acts, potentially leading to unintended consequences or reduced user trust in the technology.

Can a solution arise to the problem of distinguishing between a ‘mere imagining’ vs ‘an intended imagining to act’?

## SECTION 3: OBJECTIONS, RESPONSES & FUTURE SCOPE

### The vivacity objection

At this point, an objection may rise. One may argue that the neural patterns akin to Xβ exhibit a level of ***vivacity***^12^—a heightened intensity or clarity of neural signals—and qualify above a defined threshold, meaning the BCI would preferentially interpret and act on Xβ rather than Xα. However, there is no empirical basis for assuming that signal **strength** reliably indexes genuine intention: motor-imagery vividness varies dramatically across individuals ([27, 28]), and no study has isolated a neural marker that cleanly separates mere simulation from volitional commitment [17, 20].

Response (i), Aphantasia as a Test Case:

When we go into understanding imagination better, one can say there are types of imaginations: *pictorial* and *suppositional [29]*. Involuntary mind-wandering can categorise as suppositional imagination which is forming hypothetical mental representations. And pictorial imagination contributes to forming visual mental representations. The objection would then claim that only visual mental representations allow for vivacity and thus *neural spikes* above the threshold. If pictorial imagery alone produced vivacity above threshold, then individuals with **aphantasia**—who lack voluntary visual imagery—would be unable to generate any actionable signal.

Let’s take here the case of P2: all things same as P1 except that P2 has aphantasia. In P2’s case, his contemplations and intended imagination would all fall under suppositional imagination. Therefore, studying aphantasiac users (P2) could reveal neural patterns unique to pure motor imagings, informing decoders that distinguish **Xβ** from **Xα**.

### The threshold objection

One might also argue that BCI decoders could simply set higher thresholds to privilege the most ‘vivacious’ neural patterns. The PPC offers valuable insights into motor function, goals as well as *spatial trajectories* of movements. However, in cases of paralysis, where motor function is compromised, the patient’s motor responses and *muscular feedback* deteriorates. This degradation significantly impedes our understanding of kinaesthetic movement goals and the characteristics of grasp, trajectory, and pressure. Therefore, increasing the threshold may not be fruitful to better tackle the problem of implementational control.

Response (ii), Burdens and Limits of Training:

The threshold is user specific. Each BCI user must learn the skill of communicating with their tool, which involves regulating neural activity. Farahany [26] likens this to learning a new language. Specifically, users must control both voluntary and involuntary neural activity, a process that can be both burdensome and complex. Even involuntary mental processes contribute rich contents and neural activity, adding layers of difficulty. Current training protocols, such as reinforcement learning and feedback mechanisms, typically require users to engage in prolonged sessions to develop the necessary implementational control over covert and overt actions. For instance, mirror-based neurofeedback is often used to help users visualize their brain activity in real time, enabling gradual improvement. This is a possibility that can prevent the occurrence of unintentional overt actions, but it demands an arduous training and feedback period in turn imposing heavy cognitive and emotional burdens on users [11]

### Ethical concerns

These objections and responses underscore why the **contemplation conundrum** matters: reduced implementational control undermines agency and responsibility, and the intrusion into covert mental acts erodes **privacy of thought**. The privacy of thought permits one to imagine, suppose or engage in unintentional renderings without acting overtly upon them. Additionally, extended training cycles can also affect a user’s privacy of thought. Moreover, agency of action is tied to the successful fulfilment of contents of the user’s intentions, allowing for moral responsibility. But as seen in section 1, the action is attributed to a combination of user+tool, thus again questioning **agency** of BCI-mediated movements in the context of qualifying as overt actions. Through understanding the plausibility of voluntary unintentional covert actions, addressing the problem of **reduced implementational control** becomes essential.

### Future scope

The application of my inquiry extends into the near future along three interrelated axes. First, we must employ **passive BCIs** to map the neural correlates of intention formation—leveraging non-invasive recordings and time–frequency analyses (e.g. phase–amplitude coupling in PPC and SMA) to capture nascent motor plans before overt imagery. Second, we need to investigate the **neural basis of mental imagery** by comparing typical users with those who have aphantasia; such comparative ERD/ERS studies could isolate the signatures of **suppositional** versus **pictorial** motor imagery and reveal how pure mental affordances drive BCI commands. Third, resolving the **contemplation conundrum** hinges on empirically **delineating Xα and Xβ**—identifying distinct temporal, spectral, or spatial markers (for example, alpha-phase dynamics) that separate mere contemplation from intended imagining in real time. Alongside these empirical goals, **predictive-processing** and **embodied-action** theories may provide valuable frameworks for modeling how top-down expectations and bodily contexts shape motor imagery in BCIs [30–32]. Together, these approaches could inform decoder design and adaptive training protocols, enabling BCIs to accommodate diverse cognitive profiles, strengthen implementational control, and safeguard privacy of thought—thereby addressing the ethical stakes of agency and responsibility in BCI-mediated action.

## CONCLUSION

In this paper, I have argued that under Mele’s and Searle’s accounts of intentional action, BCI-mediated movements can be **voluntary yet unintentional**—a phenomenon captured by the **contemplation conundrum**. By showing how layered motor-imagery processes (Xα) and intended imaginings (Xβ) flow indistinguishably into overt effects (Y), we see that implementational control can break down even as the user ‘attempts’ to act. This raises ethical problems for agency, responsibility, and privacy of thought in BCI use.

## GLOSSARY

**Affordance**: opportunities or possibilities for action provided by external stimuli to an agent.

**Agency**: the capacity of individuals to act through volition.

**Aphantasia**: a condition characterised by the inability to voluntarily visualise mental imagery.

**Control:** in the ethical aspect, this refers to capacity to direct one’s actions consciously and the accountability for those actions’ consequences.

**Covert**: actions or processes that are not openly displayed.

**Imagery**: mental visualisation of things that are not present.

**Mental Action**: something you *do* mentally, like voluntary thinking, imagining.

**Metacognition**: awareness and understanding of one’s own mental states.

**Modality**: the type or mode in which something exists or is experienced or expressed, eg: visual or kinaesthetic.

**Motor Imagery**: the process of mentally simulating movement without physical execution.

**Muscular feedback**: sensory feedback from muscles, providing information about muscle tension and movement to the brain.

**Neural spikes**: Brief, synchronized increases in the electrical activity of neurons, often associated with the transmission of information across synapses.

**Overt**: Actions or behaviours that are open and observable by others.

**Pictorial Imagination**: The mental visualization of images or visual scenes.

**PPC** (Posterior Parietal Cortex): A region of the brain involved in integrating sensory information and important for spatial reasoning and motor planning.

**Responsibility**: obligation to act correctly; involves moral accountability.

**Spatial Trajectory**: in this context, neural activation across brain regions during spatial reasoning and imagination, involving key areas such as the hippocampus and parietal lobes.

**Suppositional Imagination**: the mental process of hypothesizing potential scenarios; note aphantasiacs engage in this type of imagination as they cannot form pictorial imagination.

**Threshold**: The minimum level of stimulation required to trigger a neural response or action potential in a neuron.

**V1** (Primary Visual Cortex): The part of the cerebral cortex that receives and processes visual information.

**Vivacity**: this refers to the degree of clarity and liveliness with which mental images are experienced.

## Supplementary Material

Review_history_for_OXFNSC-2024-004_kvaf002

## Data Availability

No new empirical data were created or analysed for this study. As this manuscript is a theoretical and conceptual analysis of brain–computer interface tool use, there are no original datasets to share. Any referenced datasets or published results cited herein can be accessed via their respective publications or repositories.

## Footnotes

1 Joelle Proust [2] defines mental actions as ‘the capacity for reaching specific desirable mental states through an appropriate monitoring of one’s own mental processes’. Note that in adopting her views on mental acts, I also avoid the problem of causal infinite regress and access puzzle. (for more, read A Plea for Mental Acts, [2]).

2 When I use the term visible here, I refer to BCI-mediated ‘public’ performed on a computer or external device. Paradigmatically, one uses overt actions to describe visible bodily movements of an agent. But in this case, especially for P1, there are no bodily movements, therefore I prefer the use of ‘visible’ or ‘public’ movements for BCI users.

3 Please note that a glossary of specialised terms used in this paper is provided at the end of this document for reference.

4 Note, I am **not** referring here to ‘whether’ the patient acts intentionally or not, I am referring to ‘how’ the patient wills to carry out the intention in performing the action. This will get clearer in section 2.

5 McClelland [15] explains affordances as opportunities for action. Most affordance theorists locate these in the environment (e.g. a mug’s ‘grippability’). **Mental affordances** are similarly opportunities for **mental acts** (See McClelland, Tom (forthcoming). The Mental Affordance Hypothesis. Mind.)

6 John’s intention (a mental state) resides in the flipping of the switch (an overt act)

7 See section 1.5 :Dual-content intentions X and Y in P1s case.

8 See section 1.5 :Dual-content intentions X and Y in P1s case.

9 On adopting Searle’s view, I must explain here what I interpret to be a ‘successful’ intentional action. This success comes from fulfilling one or all intentions present in the action. If one intention is not fulfilled but another is, this would still mean it is an action, but an unintentional one, at least in the capacity of not fulfilling the contents of one intention. For the action to be successfully intentional, all contents of all existing intentions must be fulfilled. The fulfilment of a prior intention could make for a voluntary action, but the unfulfillment of another underlying intention would lead to unintentional voluntary action. In such cases, moral responsibility would fall upon the agent as the action still counts as voluntary, albeit unintentional.

10 Note that following from Proust’s [2] account of an intentional mental action, if the following is satisfied – particular mental events/states/dispositions obtain as a causal consequence of the corresponding belief and desire – then it qualifies as intentional content of a mental action.

11 The ‘threshold’ denotes the system’s set criteria for recognizing actionable neural activity.

12 In this context, ‘vivacity’ refers to the distinct neural signal strength or prominence.
