# Supplementary material for: Brain-Computer Interface tool use and the Contemplation Conundrum: a blueprint of mental action, agency, and control
Source: Oxf Open Neurosci. 2025 Jun 11;4:kvaf002. doi: 10.1093/oons/kvaf002 (PMC12199723; doi:10.1093/oons/kvaf002)
Supplement: Review_history_for_OXFNSC-2024-004_kvaf002 [file review_history_for_oxfnsc-2024-004_kvaf002.docx]

Review history for OXFNSC-2024-004

**Reviewer 1 – Reject**

The author argues that some BCI-generated movements qualify as unintentional overt action, which calls for ethical considerations.

I am surprised by the chosen framework: Searle’s variant of the Causal Theory of Action, based on prior intentions and intentions in action, which strikes me as somewhat old-fashioned, and –importantly– seems the least potent of all the frameworks that could have been chosen.

Embodied perspectives would emphasise ongoing control processes during an action, which is likely to be limited in BCI cases, providing ample opportunity to compare the two types of actors. But when adopting this framework, the strict causal direction from prior intention to intention in action to action is no longer tenable, which makes it incompatible with Searle.

Enactive perspectives would further exemplify this continuous interaction between brain, body and world, and add a sense-making through action on top of that. Also this perspective would allow for discussing interesting differences between typical and BCI action

Predictive processing theories, e.g. free-energy-principle, state action is a way to solve prediction error. On these theories, typical action also consists of imagining the desired outcome. This would render the two types of actors similar, but there is perhaps differences to be found in terms of the ongoing prediction errors (like with the embodied perspective).

I believe that by using any of these three frameworks an interesting comparison could be made. Instead, the author chose to address this question using Searle’s framework. This, and all the similar CTA models (e.g. Pacherie, Bratman, Brozzo, Mylopoulos, Butterfill & Sinigaglia) are notoriously incapable of solving ‘the interface problem’: how do mental states cause motor states, given the fact that the two are categorically different in terms of nature (propositional vs we-don’t-know-what-but-not-propositional), content, direction of fit, etc. This inability lies directly at the core of the proposed comparison, rendering the analysis vulnerable.

In the current proposal, a lot hinges on the phenomenological differences between BCI and typical action, and that ‘imagining an action’ in a BCI-context is categorically different from the mental side of performing a simple action. I genuinely wonder to what extent this is indeed the case. To my knowledge of BCI -which is admittedly limited- users do not imagine the chess piece moving by themselves, in which case the BCI would have to rely on occipital signals, but imagine that they move the piece, allowing the BCI to pick up motor signals. If that is indeed the case, can we still state that the two types of action are (on the mental or phenomenological side) radically different?

Therefore, a comparison of the proposed kind calls for a detailed analysis of the phenomenology in the two situations (BCI and typical action). The manuscript does not provide such an analysis. Alternatively, one could look at the nature of the signal that is picked up by the BCI, perhaps run a principal component analysis on them, and analyse the underlying neural circuitry, and base the comparison on that. Without either, I feel that the reader Open Neuroscience learns very little from the analysis.

**Reviewer 2 – Major Revision**

The author makes a really interesting and timely point: given the way in which some BCIs work, it's difficult for the BCI to distinguish between an outcome that the subject is merely contemplating/imagining and one that they intend to perform. Because of this, when contemplating different action possibilities in the context of BCI use, one might end up performing an action they didn't intend to perform. In short, as the author says, "with BCI users, the implementational form of control is rather reduced due the action being performed as a combination of the mental act of imagining + BCI-mediated movement."

There is also another issue, however (and I'd like to hear more about whether and how this connects to the above): when planning a movement, someone using (at least certain kinds of) BCI will not be able to directly control a bodily movement (the chess-playing example at the end of p. 8 makes the point very clearly).
These are important considerations that also raise ethical issues, just as the author states.

Many subsections of the paper (up until and including section 1.4) are very difficult to follow, partly because they try to cover all the following distinctions:
- intentional vs. unintentional
- voluntary vs. involuntary
- covert vs. covert
- mental processes vs. doings
- prior intentions vs. intentions in action

sometimes with inaccuracies that are listed in the more specific comments below.
To reiterate, I take the main point to be the following: although there is a clear conceptual distinction between imagining an action and intending to perform an action, it's not clear that BCIs can make that distinction. So current BCI technology can enable subjects to perform involuntary actions simply on the basis of their subjects' contemplating / imagining different courses of action.
Assuming that this is indeed the main point, it doesn't seem to me to require many of the above distinctions. I therefore suggest a radical thinning down of the paper up until and including section 1.4, also to make it easier to follow.
More specific comments are listed below.

---p. 2
- footnote 2: in the light of the author's characterisation, "public" or "visible" would be clearer than "overt"
- The original reference for the notion of basic action is Danto, A. C. (1965). Basic actions. American Philosophical Quarterly, 2(2), 141-148.
- I'm not sure what the expression "cul-de-sac" means in this context. Could there be a clearer way to express the idea?

p. 3
- I'm not sure whether Searle or Mele ever use the expression "intentional theory of action". It's fine if this is an idea that the author has come up with, but, if so, it would be better to state more clearly that this is not being ascribed to either Searle or Mele.
- "Such a theory advocates for moral responsibility on the agent’s intention as opposed to their bodily movements [...]." This part of the sentence should be modified. It doesn't sound right that either an intention or a bodily movement could have moral responsibility. Maybe the author means that it's the intention, rather than its outcome, that has a main role in determining whether the agent has moral responsibility for their action.
- footnote 5. The difference between different kinds of BCI sounds really interesting and relevant to the article. It would be good to hear more about this.
- "According to law and policy: agency and responsibility of actions is assessed through overt actions, i.e., visible actions that cause changes in the external world; they are paradigmatically associated with bodily movements, like raising an arm, or even picking up a pen. Whereas covert actions are reserved for mental processes or mental acts, these actions are not visible externally and can account for mental processes and mental actions, including intentions."
This is potentially misleading as it almost sounds like it's only overt actions that matter for law and policy, whereas it matters even from the point of view of the law whether there was an intention to do something (think of the difference between killing with or without intention). Also, it's not obvious that covert actions can account for intentions (although this partly depends on what is meant by "account" here). The author may well be right that covert actions do not matter from the point of view of law, but they should say more about the notion of covert action (or else they can drop that distinction).

p. 4
- Shepherd (2015) does not use the term "executory control".
- "When an action is performed without a purpose, this is unintentional action. For instance, John intends to brighten the room, so he flips a switch, but instead the heating is turned on because he presses the wrong switch. Him turning on the heating is unintentional, but still a voluntary action, nonetheless. This is where things get interesting. Because both voluntary and involuntary actions could be either intentional or unintentional. John did push the switch voluntarily, but the outcome was not what he intended so (in the sense I define such an action) his turning of the heating was not intentional." Many philosophers of action would describe this situation in terms of an action only being intentional under some description, rather than saying that it is an unintentional voluntary action: John's action is intentional under the description "flipping the switch" and "turning on the light", but not under the description "turning on the heating".

pp. 4-5
- More could be said about overt vs. covert actions (see general comment above, though).

p. 7
- "an intentional action is intentional iff the contents of that intention are satisfied": The first "intentional" should be removed. This formulation should be completed by something like "in the right way", so as to avoid the problem of deviant causal chains.
- "According to Searle, an action is causally self-referential to the contents of its intentions." According to Searle, it's intentions, not actions, that are causally self-referential. Searle's notion of the causal self-referential character of intentions should be explained better, or left out. It's not clear to me whether the author needs this notion.

p. 13
- "This advocates for an action as a combination of mental processes + doings as in section 1." This feels too quick, more explanation would be useful.

- line 523: why? It seems to me as though "content of mental state to contemplate potential moves (d3 or d4) and outcomes" and "content of imagining the move to d3, the intended move" overlap. (Unless contemplating and imagining are different, but there needs to be an explanation of this.)

**Decision Letter**

| **Subject:** | Oxford Open Neuroscience - Decision on Manuscript ID OXFNSC-2024-004 |
| --- | --- |
| **Body:** | 28-Nov-2024  Dear Ms. Mehta,  Thank you for submitting Manuscript ID OXFNSC-2024-004 entitled "Brain-Computer Interface Tool Use and The Contemplation Conundrum: a blueprint of mental action, agency and control" to Oxford Open Neuroscience.  Your manuscript has been seen by two reviewers with relevant expertise and I have read it myself. The reviews can be seen below. Both reviewers find this work potentially interesting but they also suggest additional perspectives to be considered and/or clarifications.  I am therefore inviting you to submit a revised manuscript that responds to these comments.  Reviewer 1 suggests that it would be useful to consider your arguments in terms of alternative theoretical frameworks of intentional action such as embodied, enactive, or predictive processing approaches. This reviewer also suggests discussion of the phenomenology of BCI vs typical action, and how this relates to the argument you develop. Please add some discussion of these points.  Reviewer 2 has several useful suggestions for edits with a focus on clarity. I shared this reviewer’s concerns about clarity so I encourage you to consider these points carefully, however I will leave it to you to decide exactly which elements (if any) of your argument to remove. |

**Author’s Response**

Thank you for the detailed feedback on my manuscript, "Brain-Computer Interface Tool Use and The Contemplation Conundrum: a blueprint of mental action, agency and control" (Manuscript ID OXFNSC-2024-004). I appreciate the thoughtful and constructive comments from both reviewers, as well as your own insights. I have carefully considered all suggestions and revised the manuscript to address the concerns raised. Below, I outline how the revisions respond to the feedback provided.

1. Reviewer 1's Comments:
1.1 Alternative Theoretical Frameworks:
I have expanded the manuscript to incorporate discussions of embodied action theory (Gallagher, 2017) and predictive processing theory (Clark, 2013). These perspectives are applied to BCI-mediated actions to enrich the analysis of agency and control.
For clarity, a real-world example has been included to demonstrate how the embodied action theory interprets BCI actions as an integration of physical, social, and sensory contexts.
1.2 Phenomenology of BCI-Mediated Action:
I have included a discussion of phenomenology (Gallagher & Zahavi, 2008) to address the lived experience of BCI users. This addition explores how phenomenological insights into agency and ownership can enhance the ethical and practical dimensions of BCI design and use.

2. Reviewer 2's Comments:
2.1 Clarity:
I have reviewed the manuscript thoroughly to improve clarity and accessibility. Redundant passages were removed, and complex concepts (e.g., "vivacity and threshold objection") have been rephrased and supplemented with examples for greater precision.
The flow between sections has been refined to ensure logical progression of arguments.
I have also addressed all of Reviewer 2’s in-text comments, ensuring that each suggestion has been implemented to improve the clarity and coherence of the manuscript.
2.2 Ethical and Practical Challenges:
I have strengthened the discussion of implementational control, using examples of current training protocols and proposing improvements such as adaptive AI and virtual environments to reduce user burden.
Privacy concerns have been addressed with specific safeguards, including encryption, consent mechanisms, and neural data access controls, balancing ethical concerns with practical functionality.
2.3 Supporting Neuroscientific Context:
The manuscript now includes references to empirical studies (e.g., Aflalo et al., 2015; Schurger et al., 2012) to substantiate claims about neural correlates of imagination and intention.

3. General Improvements:
The manuscript has been reorganized for better coherence and readability.
A “Future Scope” section outlines key areas for further research, including the role of passive BCIs, gamma wave neuroimaging, and the neural correlates of intention formation.

I believe these revisions have strengthened the manuscript significantly, and I hope it now meets the standards of Oxford Open Neuroscience. I sincerely appreciate the reviewers’ and your valuable input, which has been instrumental in improving the quality of this work.

Thank you again for the opportunity to revise my manuscript.

Best regards,
Dvija Mehta

**Revision 1**

**Reviewer 3 – Accept**

This paper explores a set of complex questions situated at the intersection of ethics, philosophy, and the cognitive science of action, with particular attention to neuroprosthetics and brain-computer interfaces (BCIs). The central research question the author seeks to address revolves around the concepts of intention and agency in the context of BCI-mediated actions. The key thesis put forward is that certain movements facilitated by BCIs can be classified as unintentional overt actions, despite being voluntary under certain descriptions.
Throughout the paper, the author not only delves into various related ethical considerations but also engages with the well-established issue of the neural correlates of intentions. This issue touches on the degree to which the brain’s activity can be directly linked to conscious intentions, especially in the context of actions mediated by BCIs. The author’s discussion effectively integrates insights from multiple fields, including philosophy of action, cognitive science, and ethics, raising pertinent questions about the nature of agency in BCI users.
One of the most compelling aspects of the paper is its exploration of whether a BCI user truly exercises control over their actions, or whether their movements are influenced, if not determined, by the BCI itself. In doing so, the author highlights significant ethical implications related to autonomy, responsibility, and the potential for manipulation or coercion in the use of these technologies. Overall, my judgment is that the author successfully brings together interdisciplinary perspectives to address these critical issues, offering a thoughtful analysis of the complex relationship between intention, agency, and the use of BCIs.

**Decision Letter**

Dear Ms. Mehta,

Thank you for submitting your revised manuscript OXFNSC-2024-004.R1 entitled "Brain-Computer Interface Tool Use and The Contemplation Conundrum: a blueprint of mental action, agency and control".

Neither of the original two reviewers was available, so your manuscript was reviewed by a fresh reviewer who has provided helpful comments. As you will see below, this reviewer had a positive evaluation of your manuscript. However, based on my own reading of the manuscript alongside the original reviewer comments, I still believe that further revision is required before this work can be formally accepted in Oxford Open Neuroscience.
 
I have two main requests for revisions. The first relates to clarity of expression. In my comments below I have highlighted specific phrases or sentences that are hard to follow or ungrammatical, but I suggest that you carefully proofread the entire manuscript to improve clarity and to make sure that your points, especially empirical assertions, are properly backed up with evidence or citations.
 
My second main request relates to the scope of the manuscript. I take your core argument to be that at least under some theories of intentional action, users of BCIs may produce actions that are both voluntary and unintentional, which raises ethical concerns. As noted by the original reviewer 2 and by the new reviewer of this revision, this is an interesting argument and I agree with their judgement that it could make a useful contribution to the literature, as long as you include the caveat that your arguments do not necessarily hold under all theories of intentional action (as noted by the original reviewer 1). However, you also make several other arguments that are much broader in scope, including recommendations for the design of BCI systems, empirical claims about the neuroscience of imagery, and the assertion that usage of BCI systems must now be discontinued except for a limited set of cases involving disability. I do not find these claims to be well supported.
 
I think you have two options here. You could either refine these broader arguments to back them up more fully, in which case your manuscript would need to be re-reviewed by reviewers with relevant expertise in the design and ethical regulation of BCI systems, and the neuroscience of imagery. Alternatively, you could perform a “radial thinning down of the paper” (in the words of the original reviewer 2) to focus on your core argument only. My recommendation would be to opt for the latter approach. If you choose to follow this course, I will endeavour to make a decision on your manuscript without further review.
 
Specific comments:
 
p. 6 “Many philosophers of action make this distinction”. This needs to be backed up with one or more references.
 
p. 6 “When one does something by means of doing something else, for instance, John waves at his friend.” This sentence is ungrammatical.
 
p. 7 “semantic rich” -> “semantically rich”, “lead to complexity” -> “leads to complexity”
 
p. 9 “Another example is John flipping a light switch to brighten a room exemplifies an intention-in-action”. Ungrammatical.
 
p. 10 “Applying this to the P1 case, a BCI-integrated individual who intends to play chess on his computer (see Fig 2 below).” Ungrammatical.
 
p. 12 “When it comes…” This paragraph is repeated twice.
 
p. 14, and several other points in the manuscript: “neuro-scientifically” -> “neuroscientifically”
 
p. 15 “This is because Imagination activates motor imagery involving both visual and kinesthetic modalities”. This needs to be backed up with one or more citations. Also, Imagination should not be capitalised, kinesthetic -> kinaesthetic. 
 
p. 16, and several other points in the manuscript: “neural spikes”. I think “neural activity” would be more appropriate. Also, the paragraph in which this occurs contains multiple empirical claims, each of which needs to be backed up with one or more citations.
 
p. 16 “So far as all we know” -> “So far as we know”
 
p. 16 “differentiating between when a mental process is an ‘intention to act’, and when a mental process is a mere imagination, is nearly impossible”. Unclear what you mean by “nearly impossible”. You should either explain what you mean by this, or use less assertive language, e.g. “may be difficult or impossible”. NB this may be a good point to note the comment from the original reviewer 1, who suggests that you discuss predictive processing theories of brain function. According to these theories, the formal distinction between a prediction and an intention may collapse (cf ‘active inference’). If this is correct, it could be claimed that it will not always be possible to distinguish the neural correlates of intention and prediction. This could be taken to support the argument you are making here, so it could be useful to note at this point.
 
p. 16 “we know but a drop in the ocean when it comes to the neural correlates of intentions”. This comes across as unclear and overly colloquial. What exactly is unknown? There is certainly a large literature on this topic, so you need to either clarify your meaning or remove this.
 
p. 17 “there are no neural patterns that can separate an ‘imagination’ from an ‘intended imagination to act’”. This is a strong claim that needs to be backed up in some way, not simply asserted (as noted above, one way of making this argument could be by referring to predictive processing theories and active inference). Alternatively, you could use less assertive language, e.g. “it may be difficult or impossible in practice to find neural patterns that can separate…”
 
Section 3 onwards: From this point on, you make multiple strong empirical, ethical, and practical assertions that are not backed up by evidence or citation. You also make many claims that fall well outside the scope of the arguments that you have been considering up to this point. For the best chance that your article is eventually accepted, my suggestion is to simply remove this entire section. Alternatively, if you want to include this material you need to include much more detailed support for your claims, which would probably need to be independently reviewed by experts from the relevant academic fields.

**Author’s Response**

Dear Sam,

Thank you for your detailed feedback and for guiding the restructuring of my manuscript. In this revision I have:

Radially thinned down the scope to focus squarely on the core claim—that under Mele’s and Searle’s theories of intentional action, BCI‐mediated movements can be voluntary yet unintentional (the “contemplation conundrum”). Irrelevant empirical and policy recommendations have been removed, and broader design prescriptions have been reframed as a concise research agenda.
Implemented all in‐line editing suggestions, including grammatical corrections, clearer analytic language, and careful citation of empirical assertions. I have replaced colloquialisms, clarified terms like “mental affordances,” and ensured consistency in philosophical terminology.
Updated and expanded the reference list with recent neuroscientific studies (2020–2025), including work on motor‐imagery variability (Pfurtscheller & Neuper, 2020), aphantasia (Zeman, 2024), passive BCI intention mapping (Park et al., 2024), and phase‐dependent agency markers (Bertoni et al., 2025).
Strengthened Section 3 by removing unsupported claims, preserving the vivacity‐threshold objection, and reframing the remainder as a focused, three‐axis research agenda. I have added a brief note on complementary theoretical frameworks (predictive‐processing and embodied‐action theories) without expanding beyond the paper’s primary argument.
Tightened the abstract and conclusion to avoid repetition and to underscore the paper’s ethical stakes—implementational control, agency, and privacy of thought—while pointing clearly to the next steps in empirical research.

Regards,

**Revision 2**

**Decision Letter**

Dear Ms. Mehta,

Thank you for submitting your very responsive revision, Manuscript ID OXFNSC-2024-004.R2, entitled "Brain-Computer Interface Tool Use and The Contemplation Conundrum: a blueprint of mental action, agency and control".  

I'm pleased to say that I am happy to accept this manuscript in principle, however I am issuing a "Minor Revision" decision because I would like to request four small edits before the manuscript can be formally accepted:

Page 16: "Nothing neuroscientific distinguishes one pattern of motor imagery from the other...". This seems too strong, seeing as future discoveries in neuroscience might conceivable identify a suitable neuroscientific marker. I suggest that you begin this sentence with something like "No known neuroscientific marker distinguishes one pattern of motor imagery from the other..." to improve clarity.

Page 18: "it may instantiate Xα+Y, yielding a voluntary but unintentional overt action, rather
than the desired Xα+Y". Please check. Should the second Xα+Y actually be Xβ+Y?

Pages 20: "such comparative ERD/ERS studies will isolate the signatures". Page 21: "there approaches will inform decoder design". Both of these seem to prejudge the outcome of empirical work, I suggest that you replace "will" with "could" in both cases.

If you are willing to make these edits, I will aim to act swiftly to pass your manuscript to production.

**Author’s response**

Dear Professor Gilbert,

Thank you for your thorough review and for accepting my manuscript in principle. I’ve made all four of the requested edits:

Updated the Page 16 sentence to “No known neuroscientific marker distinguishes one pattern of motor imagery from the other…”
Corrected the Page 18 term to “Xβ + Y” in place of the second “Xα + Y.”
Replaced “will” with “could” on Page 20 (“Such comparative ERD/ERS studies could isolate the signatures…”).
Replaced “will” with “could” on Page 21 (“…these approaches could inform decoder design.”).
In addition, I conducted a final proofreading pass to ensure consistency and clarity throughout. The revised manuscript is attached in both pdf and docx format.
Please let me know if there’s anything else you’d like me to address. Thank you again for your time and guidance.

Best regards,

**Revision 3**

**Decision Letter**

| **Subject:** | Oxford Open Neuroscience - Decision on Manuscript ID OXFNSC-2024-004.R3 |
| --- | --- |
| **Body:** | 04-Jun-2025  Dear Ms. Mehta,  It is a pleasure to accept your manuscript entitled "Brain-Computer Interface Tool Use and The Contemplation Conundrum: a blueprint of mental action, agency and control" in its current form for publication in the Oxford Open Neuroscience. |
